# The Medical Students’ Manual of Chemistry

**Published:** 1890-12

**Authors:** 


THE MEDICAL STUDENTS' MANUAL OF CHEMISTRY. By R. A. WITTHAUS, A. M., M. D., Professor of Chemistry and Physics in the University of the City of New York; Professor of Chemistry and Toxicology in the University of Vermont; Member of the Chemical Societies of Paris and Berlin; Member of the American Chemical Society; Fellow of the American Academy of Medicine, of the New York Academy of Medicine, of the American Association for the Advancement of Science, etc. Third edition, pp. xii.—528. William Wood & Company, New York. 1890.

When we consider how much dross and chaff is placed upon the market under the title, Text-book of Chemistry, it is indeed a pleasure to occasionally meet a work of such value as the one before us. We extend to Professor Witthaus our heartiest congratulations for his success in the arrangements of this manual. It is clear, concise, brought down to date, and sufficiently extensive to give medical students a thorough groundwork in the grand science of chemistry.

Part I. Introduction occupies fifty-two pages, divided into three general groups: General Properties of Matter; Physical Characters of Chemical Interest; and Chemical Combination. In the last group, the elements, their combination; the atomic theory, atomic and molecular weights; atomicity; symbols; acids, bases, and salts; stoichiometry; nomenclature; radicals, composition and constitution; and classification of the elements are all carefully considered. The author adopts the following classifications:

Class I. Typical elements.

Class II. Elements whose oxides unite with water to form acids, never to form bases, which do not form oxysalts.

Class III. Elements whose oxides unite with water, some to form bases, others to form acids, which form oxysalts.

Class IV. Elements whose oxides unite with water to form bases, never to form acids, which form oxysalts.

Within the classes a further subdivision is made into groups, each group containing those elements within the class which have equal valences, which form corresponding compounds, and whose *chemical characters* are otherwise similar.

It will readily be seen that Class I. will contain hydrogen and oxygen; Class II., those elements commonly called non-metallic elements, or those having an electro-negative character, with the exception of oxygen. Class IV. contains all those elements that are decidedly basic or electro-positive in character, excepting hydrogen; while Class III. contains a group of elements lying between the decidedly acid or negative, and the decidedly basic or positive elements. To this class belong those elements which act, now as an acid, now as a base. The classification is an excellent one, and does not present the difficulties in the lecture-room which are experienced in the use of a classification based upon the periodic law.

Part II. is devoted to Special Chemistry, *i. e.*, to a study of the properties of the elements and their compounds.

Of Organic Chemistry we have but little to say. It is clear and concise, and the student need have no fear of its being a bug-bear.

Part III. treats of Chemical Technics, in which the student is taught something of chemical manipulation.

The term "hydrate" is used for such compounds as NaOH. In this we must say that we differ with the author as to the advisability of using this term for compounds of this class. We believe the term "hydroxide" better adapted, and believe that "hydrate"

should be reserved to characterize such compounds as unite with water without the evolution of hydrogen; for example, sulphuric acid hydrate, having the composition $H_2SO_4+H_2O$. But this is a small matter.

On the whole we consider Prof. Witthaus's Manual an excellent one, and cheerfully recommend it to the profession.

The publishers have gotten the volume up in beautiful shape; the paper is good, the print clear and very easily read, and the illustrations excellent. J. A. M.

